# The science behind TCM and Gut microbiota interaction—their combinatorial approach holds promising therapeutic applications

**DOI:** 10.3389/fcimb.2022.875513

**Published:** 2022-09-13

**Authors:** Wenrui Xia, Bei Liu, Shiyun Tang, Muhammad Yasir, Imran Khan

**Affiliations:** ^1^ Hospital of Chengdu University of Traditional Chinese Medicine, Chengdu, China; ^2^ Chengdu University of Traditional Chinese Medicine, Chengdu, China; ^3^ National Drug Clinical Trial Agency, Teaching Hospital of Chengdu University of Traditional Chinese Medicine (TCM), Chengdu, China; ^4^ Special Infectious Agents Unit, King Fahd Medical Research Center, King Abdulaziz University, Jeddah, Saudi Arabia; ^5^ Department of Biotechnology, Abdul Wali Khan University Mardan, Khyber Pakhtunkhwa, Pakistan

**Keywords:** Chinese medicine, TCM, gut microbiota, prebiotics, TCM-microbiota interaction, TCM-bacteria interaction, medicine

## Abstract

The trend toward herbal medicine as an alternative treatment for disease medication is increasing worldwide. However, insufficient pharmacologic information is available about the orally taken medicines. Not only herbal medicine, but also Western drugs, when passing through the gastrointestinal tract, interact with trillions of microbes (known as the gut microbiome [GM]) and their enzymes. Gut microbiome enzymes induce massive structural and functional changes to the herbal products and impact the bioavailability and efficacy of the herbal therapeutics. Therefore, traditional Chinese medicine (TCM) researchers extend the horizon of TCM research to the GM to better understand TCM pharmacology and enhance its efficacy and bioavailability. The study investigating the interaction between herbal medicine and gut microbes utilizes the holistic approach, making landmark achievements in the field of disease prognosis and treatment. The effectiveness of TCM is a multipathway modulation, and so is the GM. This review provides an insight into the understanding of a holistic view of TCM and GM interaction. Furthermore, this review briefly describes the mechanism of how the TCM–GM interaction deals with various illnesses.

## 1 Introduction

Traditional Chinese medicine (TCM) is a medicinal system that is thousands of years old and has been widely adopted for treating diseases. The precarious therapeutic approach of Western medicine has limited TCM application to the Chinese people only. To make TCM a good competitor with Western medicine, the Chinese government has established 16 TCM centers to modernize TCM application (Feng et al., 2017). Toward the moderation of TCM, OMICS is one of the imperative fields of research that can search for potential targets on which TCM acts and trigger downstream signaling cascades (Joshi et al., 2010).

Unlike Western drugs, TCM holistically improves body physiology against diseases and, therefore, is prescribed for holistic characterization of the patient’s syndrome by following the yin and yang and five elements theory, visceral meridian theory, etiology and pathogenesis theory, diagnosis, and therapy theory (Huang K. et al., 2021). TCM can be available in the form of decoction, powder, pill, and paste. Interestingly, the same ingredients can compose a prescription; however, different dosages can have distinct functions.

A bigger part of TCM is orally taken, which then passes through physiological changes, mainly, through the enzymatic activities that are secreted by trillions of gut-residing microbes (known as gut microbiota [GM]) and host cells. These activities can remodel the functional constituents of TCM. Importantly, GM display a critical role in host health even in the occurrence of disease. It is estimated that GM constitutes about 43% of the human body by cell count and encodes 100 times more genes than our body genes (Knight et al., 2017). According to the latest statistics, the human microbiome encodes 2-20 million genes, surpassing ∼20,000 human genes (Knight et al., 2017). These microbial genes are presented to hosts for various functions, including digestion, metabolism, and immune system maturation (Cani, 2009). The human GM (approximately 99%) is composed of bacteria. And, among the body parts, the gastrointestinal tract is more densely populated. These commensals inhabit the human gut with a magnitude of about 100 billion to 1 trillion bacterial cells in one gram of human stool (Hugon et al., 2015). A balanced GM is key to host health, and any dysbiosis in gut microbial composition could put the host at a risk for obesity, inflammatory bowel disease, diabetes, autism, rheumatoid arthritis, and colorectal cancer (CRC) (Musso et al., 2010; Stefka et al., 2014; Sun and Kato, 2016; Maeda and Takeda, 2017).

Most importantly, TCM–GM research is updating our understanding of disease prognosis and treatment. A growing trend is developing among TCM researchers to define the pharmacology of orally taken TCM by harnessing the potentials of the GM. TCM interacts with trillions of gut-residing microbes and their enzymes that present massive structural and functional changes. These microbes can affect the bioavailability and efficacy of the herbal therapeutics. Not only herbal medicine, but also the efficacy of Western medicines relies on the GM. For instance, the efficacy of cyclophosphamide, an anticancer immune-suppressant drug, is dependent on two intestinal commensals called *Enterococcus hirae* and *Barnesiella intestinihominis* (Viaud et al., 2013). This interaction also unmasks the holistic therapeutic approach of TCM and reveals the vital role of GM. With a growing understanding of TCM–GM, various GM-based therapeutic approaches are developing. However, being a new topic, there are questions that still need to be addressed: (a) How does TCM remodel GM diversity and composition? (b) How can the GM remodel TCM constituents and their function? (c) Does TCM function as a growth substrate for the GM? Nevertheless, the study of GM–TCM interaction has opened an exciting avenue for drug discovery and new drug targeting (El Kaoutari et al., 2013) (Chen F. et al., 2016). This review focuses on highlighting key achievements from TCM–GM research.

## 2 TCM–GM interaction

TCM–GM interaction employs a comprehensive approach and is making groundbreaking achievements in the field of disease prognosis and treatment. Since targeted intervention for remodeling GM composition has shown encouraging results in the field of disease prevention and treatment, in this regard, TCM has become one of the approaches through which GM composition is targeted and remodeled for predetermined therapeutic outcomes. Various TCM components, such as dietary fiber, phenolic compounds, and undigested carbohydrates, are proven to upregulate the growth of beneficial intestinal microbes, improve gut homeostasis, and alleviate disease symptoms (Makki et al., 2018). For example, several studies show GM remodeling effects of saponins, naturally occurring compounds extracted from a Chinese medicinal herb (known as *Gynostemma pentaphyllum*). Saponins are reported to facilitate the growth of beneficial bacteria and suppress cachexia-like symptoms in mouse models (Chen L. et al., 2016; Huang et al., 2017).

Not only does TCM affect the GM composition and diversity, but TCM therapeutic efficacy is dependent on the presence of certain bacterial species and their enzymes. For instance, PHY906 (derived from four Chinese herbs) is an anticancer medicine that can reduce irinotecan toxicity in advanced-stage CRC patients. However, the efficacy of PHY906 is dependent on β-glucuronidase, an enzyme produced by intestinal bacteria (Lam et al., 2010). Similarly, curcumin supplements to a *Il10^−/−^* mouse promote the abundance of Lactobacillales and increase bacterial diversity that is concurrently accompanied by a reduced polyp burden (McFadden et al., 2015). Nonetheless, GM also improves the availability of TCM, which is one of the classic limitations of TCM.

**Figure 1:**
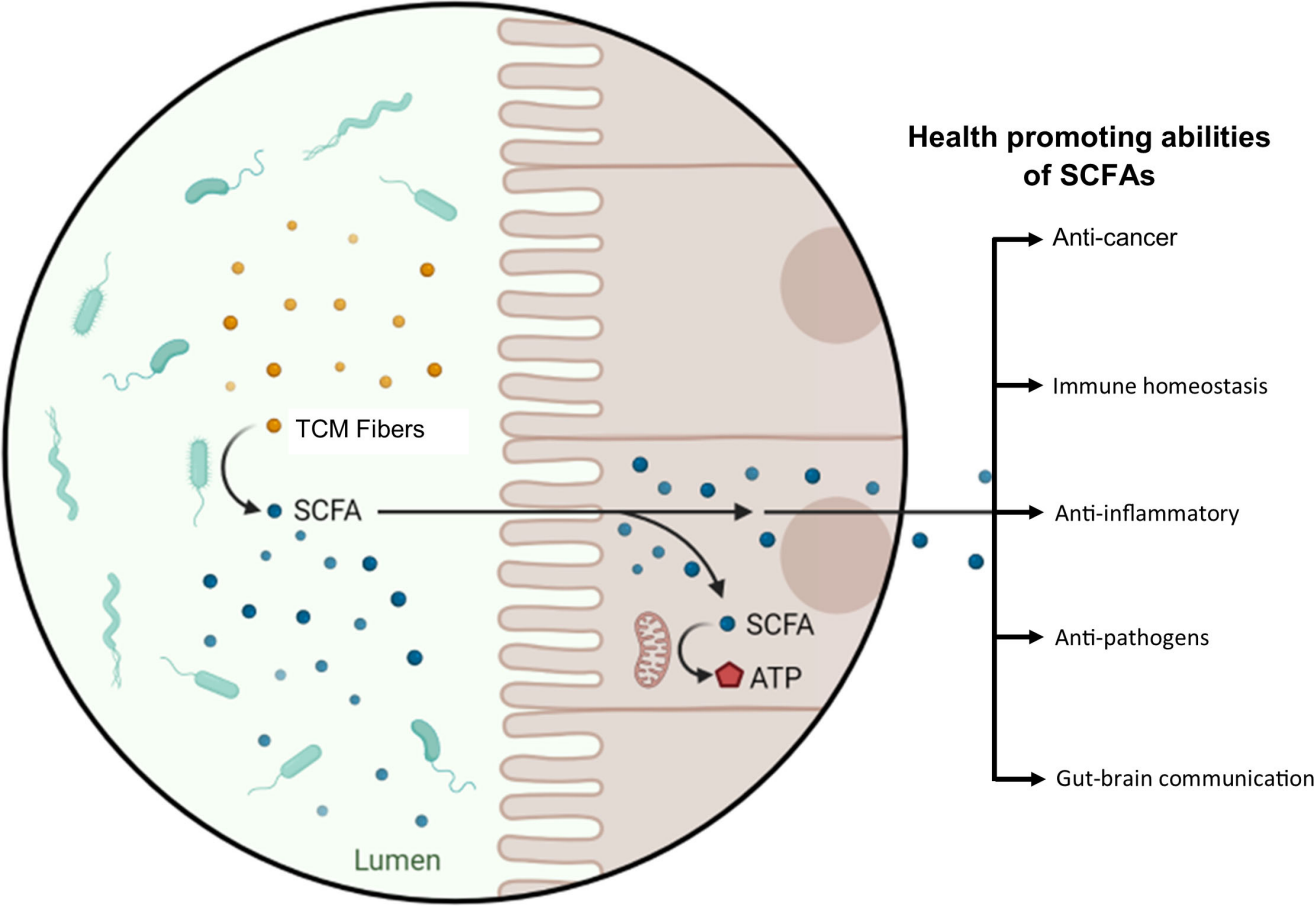
TCM–GM combinatorial therapeutic effect, taking polysaccharides as an example. Dietary and medicinal fibers are converted into SCFAs by the action of the GM. SCFAs are an energy source for colonocytes and possess other health-promoting abilities; a few of these abilities are displayed in the figure.

Another classic example of the TCM–GM interaction is the ingestion of indigested polysaccharides. Indigestible polysaccharides undergo biochemical processes in the gastrointestinal tract and are converted into short-chain fatty acids (SCFAs) by gut microbes. SCFAs are indispensable in maintaining our health, especially colon health (**Figure 1**). Nonetheless, SCFAs are the energy sources for colonocytes to guarantee the activity of the colon (Wong et al., 2006). Besides this, through inhibition of histone deacetylases and activation of G-couple protein receptors (GPRs), SCFAs can regulate the immune system and correct metabolic disorders (Sun et al., 2017). In addition, SCFAs can also contribute to the gut barrier construction *via* enhancement of the expression of the *MUC-2* gene, modulation of oxidative stress, and upregulation of tight junction molecules (Wang et al., 2012; van der Beek et al., 2017). Furthermore, SCFAs also help in alleviating metabolic syndrome in a high-fat-diet mouse model by inhibiting the expression of pro-inflammatory cytokines, such as IL-1β and IL-6 as well as toll like receptor 4 (TLR4) in adipose tissue (Zhai et al., 2019). The nexus of TCM–GM is a relatively new concept, and interests have been developing toward this field since 2015. In the following section of the manuscript, we summarize how TCM and GM influence each other directly and/or indirectly.

### 2.1 Effects of TCM on GM

#### 2.1.1 Direct effects

TCM plays a significant role in promoting the growth of intestinal probiotics and is one of the ways for TCM to exert its curative effect (**Table 1**). For instance, *Pogostemon cablin* (Blanco) Benth (PC) is a Chinese medicinal plant, traditionally used for the treatment of gastrointestinal symptoms. Four extractions of PC (*i.e.*, patchouli essential oil, patchouli alcohol, pogostone, and β-patchoulene) were found to promote the abundance of beneficial bacteria, such as *Anaerostipes butyraticus*, *Butytivibrio fibrisolvens*, *Clostridium jejuense*, *Eubacterium uniforme*, and *Lactobacillus lactis* (Leong et al., 2019). Other compounds, such as polysaccharide, also have well-known prebiotics. It is reported that the *Lycium barbarum* polysaccharides reduce the abundance of potential pathogens, such as *Allobaculum stercoricanis*, *Parasutterella excrementihominis*, and *Tannerella* spp., and enhance the abundance of beneficial bacteria, including *Clostridium* sp., *Lachnoclostridium clostridium xylanolyticum*, *Lachnoclostridium clostridium saccharolyticum*, and *Lactobacillus reuteri* in C57 mice (Xia et al., 2020b). Besides this, mushroom polysaccharides (*Ganoderma lucidum* and *Poria cocos*) can also exert beneficial effects by altering GM composition and improving the ratio of beneficial bacteria to potential pathogens (Khan et al., 2018).

**Table 1:** Herbal formula effects the GM.

Herbal formula	Typical diseases/model	Effects on GM	References
**Xiexin Tang**	High-fat diet-induced type-2 diabetic Sprague-Dawley rats	Increased phyla Proteobacteria and Actinobacteria. Elevated abundance of Alloprevotella, *Barnesiella*, *Ventriosum* group, *Lachnospiraceae* UCG-001, and *Papillibacter* was observed.	(Wei et al., 2018)
**Chaihu-Shugan-San**	High-fat diet-induced non-alcoholic fatty liver disease Sprague-Dawley rats	Decreased level of *Enterobacteriaceae*, *Staphylococcaceae* and *Veillonella* was detected whereas *Anaeroplasma* was elevated	(Liang et al., 2018)
**Coptis chinensis decoction**	Normal Sprague-Dawley rats (male)	*Acidovorax*, *Enterobacter* and *Veillonella* increased whereas *Bacteroides* and *Prevotella* were suppressed.	(Li et al., 2015)
**Dahuang-Mudan decoction**	DSS induced colitis mice	Firmicutes, Actinobacteria, *Butyricicoccus pullicaecorum* were promoted, whereas Proteobacteria and Bacteroidetes were decrease	(Luo et al., 2019)
**Gegen-Qinlian decoction**	T2D patients	Promoted abundance of *Faecalibacterium* spp., *Gemmigar*, Lachnospiracea_incertae_sedis *Escherichia*, *Parasutteralla*	(Xu et al., 2015)
**Si Miao Formula**	High fat diet induced NAFLD mouse model	Enhanced abundance of *Akkermansia muciniphila*	(Han et al., 2021)
**Huai Hua San**	Apc*^Min/+^* CRC mouse model	Promoted abundance of *Akkermansia*, *Barnesiella*, *Lachnoclostridium*, *Ruminococcus*; And suppressed *Helicobacter* species and hydrogen sulfide producing-bacteria	(Xia et al., 2020a)
**Huangqin Decoction**	UC mouse model	Increased *Lactococcus*; Decreased *Desulfovibrio* and *Helicobacter*	(Li et al., 2020)
**Zengye Decoction**	Constipated rat model	Decreased *Desulfovibrio* and *Ruminococcus*	(Liu et al., 2019)

**Figure 2:**
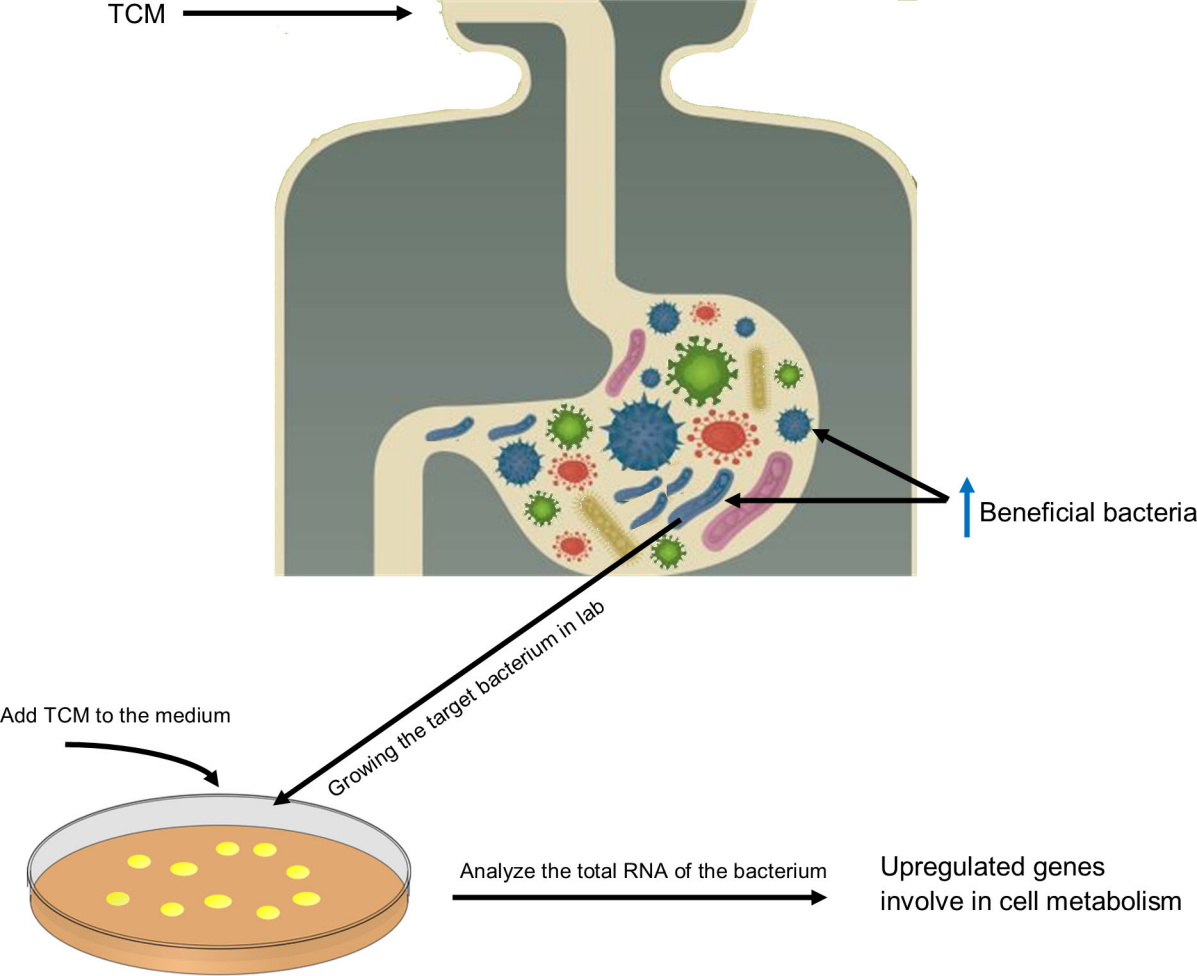
Graphic illustration of the TCM effect on GM composition and gene expression and a guide to investigate the TCM–GM interaction. This illustration shows the direct effect of TCM on GM composition. TCM intake promotes growth of certain bacteria; for instance, in the case of saponins, *B animalis* and *L. reuteri* become abundant. To check how TCM modulates the genome expression of microbes, target bacteria should be culture *in vitro* in the presence of TCM, and their growth kinetics and gene expression should be monitored. In the case of saponins, it is observed that saponins promote the expression of genes involved in metabolism and biogenesis that contribute to promoting growth of these bacteria in a habitat.

Several studies report promoted growth of beneficial bacteria after feeding a host with the saponin of the *Gynostemma pentaphyllum*. But it was unknown how saponin could promote the growth of bacteria. In a recent study, Liao et al. found out, in an *in vitro* setup, that saponin promotes growth of the beneficial bacteria (such as *Bifidobacterium animalis* and *Lactobacillus casei*) by upregulating several bacterial key genes involve in biogenesis and metabolic pathways (e.g., *gatC*, *rpmH*, *ruvA*, *yajC*, and *rsfS*) (Hugon et al., 2015). For a better understanding, **Figure 2** shows how TCM can directly impact the composition of the GM. This can also be taken as a guide to investigate the interaction between TCM and GM.

#### 2.1.2 Indirect effects

**Figure 3:**
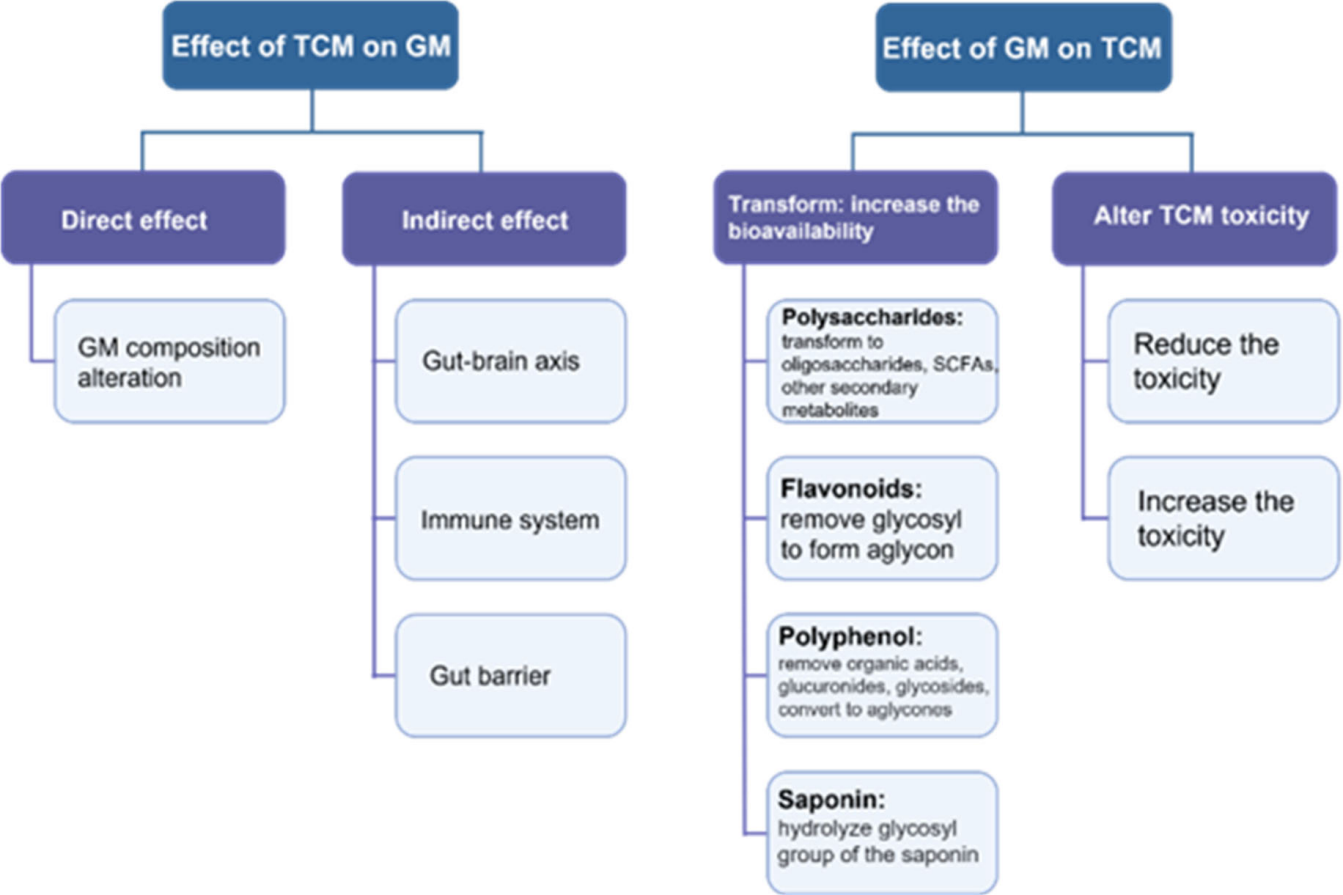
A schematic diagram displaying the interaction between TCM and GM.

Certainly, the TCM therapeutic mechanism has outcomes far beyond simply changing the composition of the GM. As mentioned above, in health maintenance and disease development, the GM exerts its function in multiple ways. In this regard, the immune system has an impactful role in both health and diseases. TCM affects the host’s immune system to secrete materials that possess GM-modulating properties (Ostaff et al., 2013). For instance, the extracts of *Codonopsis pilosula*, *Saussurea lappa*, *Imperata cylindrical* var. *major and Melia toosendan* increase the secretion of antimicrobial peptides that markedly impact GM composition (Zhou et al., 2016). In addition, TCM can also regulate GM composition and diversity by affecting the structure of the intestinal barrier. For instance, *rhubarb enema* is a common TCM medicine that can improve intestinal barrier integrity and consequently regulate GM dysbiosis (Ji et al., 2020). This TCM increases the expression of tight junction (TJ) molecules, thereby promoting the proliferation of gut epithelial cells and significantly enhancing the gut intrinsic mucosal defense, which results in the prevention of harmful substances and sequentially helps to restore GM composition (Tian., 2020). Another way that TCM promotes the growth of beneficial bacteria in the gut is stimulating the gut mucosa, which is a source of nutrients for bacteria such as *Lactobacillus* and *Akkermansia* (Tian., 2020). These bacteria have a mutual collaboration in repairing the gut barrier by promoting the growth of goblet cells and mucin 2 (Lu et al., 2021). TCM (such as herbal polysaccharides) can also regulate GM composition through the gut–brain axis (Sun et al., 2020). The direct and indirect effects of TCM on GM composition and diversity is displayed in **Figure 3**.

### 2.2 Effects of GM on TCM

#### 2.2.1 GM transforms TCM into functional metabolites

In the gastrointestinal tract, TCM exerts a curative effect through a series of complex processes, such as absorption, transformation, and metabolism. During these processes, the GM plays an important role in the modification, absorption, and detoxication of TCM ingredients and consequently improves its efficacy (Wilson and Nicholson, 2017). The GM can also contribute to the bioavailability of TCM biochemical components (Wu and Tan, 2019; Zhang et al., 2021). Below we explain and display in **Figure 3** how the GM can transform TCM into functional metabolites by taking polysaccharides, flavonoids, polyphenols, and saponin (**Table 2**).

**Table 2:** Transformation of typical TCM compounds by GM.

TCM compounds	New metabolites	Effects	Reference
Geniposide	Genipin	Increased the bioavailability	(Kang et al., 2012)
Rutin	Quercetin, 4-hydroxybenzoic acid; 3,4-dDihydroxybenzoic acid; 3,4- dihydroxyphenylacetic acid		(Kim et al., 1998)
Baicalin	Deglycosylated baicalein and methylated aglycon oroxylin A		(Trinh et al., 2010)
Naringin	Naringenin, 4-hydroxybenzoic acid, phloroglucinol, 2,4,6-trihydroxybenzoic acid, 4- hydroxyphenylacetic acid		(Kim et al., 1998)
Berberine	Dihydroberberine		(Feng et al., 2015)
Procyanidins, anthocyanins	Phenylacetic acid, mono- and dihydroxyphenylacetic acids, mono- and dihydroxyphenylpropionic acids, and hydroxybenzoic acid and protocatechuic acid		(Moco et al., 2012)
Tea polyphenols	Hydroxyphenyl-c-valerolactone		(Chen and Sang, 2014)
Protopanxadiol-type ginsenosides	Compound K and ginsenoside Rh2		(Kim, 2018)
Rhein	Rheinanthrone		(Takayama et al., 2012)
Sennoside	Sennidin		(Hattori, 1988)
Aconitine (Diester diterpene alkaloids)	Mono-ester aconitum alkaloids and lipo-alkaloids	Reduced the toxic	(Wang et al., 2015; Zhao et al., 2007)
Strychnine	16 hydroxystrychnine	Increased the toxic	(El-Mekkawy et al., 1993)
Amygdalin	Mandelonitrile		(Carter et al., 1980)

##### 2.2.1.1 Polysaccharides

A considerable portion of TCM comprises polysaccharides, and they are reported to possess prebiotic effects (Khan et al., 2018; Xia et al., 2020b). Polysaccharides possess anticancer properties (Zhang et al., 2018), antiobesity effects (Wu et al., 2019), and anti-inflammatory effects (Niu et al., 2021). Due to the limited digestive enzymes encoded in the human genome, polysaccharides remained undigested until they reach the colon (Baumann and Bisping, 1995), where they degrade into fermentable oligosaccharides, such as β-glucans, and are broken down by microbial saccharolytic machinery (Lin et al., 2021; Niu et al., 2021). Polysaccharides are also converted to SCFAs and other secondary metabolites (Niu et al., 2021). SCFAs are a source of energy for colonocytes, regulate the immune system, and correct metabolic disorders (Sun et al., 2017). In addition, SCFAs contribute to the gut barrier construction *via* enhancement of the expression of MUC 2, modulation of oxidative stress, and upregulation of TJs (Wang et al., 2012; van der Beek et al., 2017).

##### 2.2.1.2 Flavonoids

Flavonoids are important ingredients of TCM, usually combined with carbohydrates to form glycosides. Inside the gastrointestinal tract, flavonoids are converted into various by-products through the action of the GM and, hence, affect flavonoids’ health-related abilities for the host (Chiou et al., 2014). Bacteria that can convert dietary flavonoids include *Bacteroides uniformis*, *Bacteroides ovatus*, *Bifidobacterium adolescentis*, *Enterococcus casseliflavus*, *Enterococcus avium*, *Flavonifractor plautii*, *Lactobacillus plantarum* IAM, *Parabacteroides distasonis*, *Eubacterium cellulosolvens*, *E. coli* ATCC BAA-97 and several others (Braune and Blaut, 2016). These bacteria convert dietary flavonoids through several enzymatic reactions that include O-Deglycosylation, C-Ring cleavage, Reduction, O-Desmethylangolensin cleavage, and Dehydroxylation. Most of the bacteria are capable of flavonoid conversion through O-deglycosylation (Braune and Blaut, 2016).

Through GM-secreted enzymes, the glycosyl is removed to form aglycon, which can then be absorbed by the body. Bacterial enzymes, such as α-rhamnosidase, exo-β-glucosidase, and endo-β-glucosidase, transform rutin, hesperidin, naringin, and poncirin into their aglycones, which have more of an antiplatelet effect and cytotoxicity than their parental compounds (Kim et al., 1998). Trinh et al. found that the products of baicalin (a flavone glycoside) when undergoing physiological changes exerted by the GM result in deglycosylated baicalein, methylated aglycon, and oroxylin-A. It is noticed that baicalin and oroxylin-A are more potent than the parental compound (Trinh et al., 2010). Another flavanol, named kaempferol, is reported to lower the tumor burden in the host by promoting the abundance of beneficial bacterial that were involved in secondary bile acid synthesis. These changes were concurrently accompanied with improved expression of the farnesoid X receptor (FXR), a main regulator in bile acid signaling (Li et al., 2022).

##### 2.2.1.3 Polyphenol

Polyphenol, one of the most important secondary metabolites of plants, has received increasing awareness in recent years. Since most polyphenols have lower bioavailability and they reach the colon, a densely inhabited part of the gastrointestinal tract, thus, a bidirectional interaction between the GM and polyphenols commences. GM convert polyphenols to aglycones by removing organic acids, glucuronides, and glycosides (Makarewicz et al., 2021). The GM can convert polyphenols through several enzymatic processes to various metabolites. For instance, enterolactones are produced from lignans, equol is produced from daidzin, and urolithins from ellagitannins (Espín et al., 2017). Contrarily, polyphenols can also affect GM composition by promoting the growth of beneficial bacteria, such as *Akkermansia muciniphila*, *Lactobacillus reuteri*, *Lactobacillus acidophilus*, and *Faecalibacterium prausnitzii* (Cueva et al., 2017; Espín et al., 2017; Gowd et al., 2019; Liu et al., 2020). In addition to the growth-promoting abilities of polyphenols, these compounds are also antimicrobial in nature and can inhibit the growth of bacteria, mostly potential pathogens (Rodríguez-Daza et al., 2021).

Besides this, microbes also produce phenolic metabolites that possess antioxidant, anti-inflammatory, and antiproliferative activities (Saha et al., 2016). This metabolite production is dependent on the (poly)phenol-associated enzymes produce by the GM. Interindividual differences of the GM are connected to different metabotypes, which are related to different health outcomes in people after taking polyphenol (Cortés-Martín, 2020). In short, among the gut-dwelling bacteria, it is beneficial for the host to inhabit (poly)phenol-degrading bacteria in the gut that could ensure the bioconversion of polyphenols and enhance the host’s health.

##### 2.2.1.4 Saponin

Saponins are markedly studied and practiced in TCM for various therapeutic purposes. More recently, it has been observed that the therapeutic ability of saponin, at least partly, is through improving GM composition (Chen L. et al., 2016; Huang et al., 2017; Khan et al., 2019; Xu et al., 2020). As with several other TCM constituents, the absorption rate of saponin in the human body is exceedingly low, and therefore, the bioconversion of saponins through microbes (such as *Aspergillus* sp., *Bacillus* sp., and lactic acid–producing bacteria) is gaining popularity. Microbes mainly hydrolyze the glycosyl group of the saponin. For instance, protopanaxatriol ginsenosides are hydrolyzed into G-Rh_1_ and G-F_1_ when interacting with the GM in the gut (Tawab et al., 2003).

The gut-residing microbes harbor a variety of enzymes that metabolize saponin into by-products. For instance, *Bifidobacterium*, *Bacteroides*, and *Prevotella* species encode α-arabinopyranosidase, β-glucosdiase, and α-arabinofuranosidase enzymes that can cleave the sugar moiety and hydrolyze Protopanaxadiol-type ginsenoside into monoglucosylated ginsenoside compound K (Karikura, 1992; Hasegawa et al., 1996; Park et al., 2000; Eun-Ah Bae, 2002). In addition, *B. adolescentis* and *L. rhamnosus* are also reported for the bioconversion of saponin in the host gut (Wang et al., 2021). A pharmacokinetic study through an oral treatment with a ginseng saponin fraction confirmed that ginsenosides Rh1, F1, and compound K are the metabolites for parental ginsenosides by GM (Tawab et al., 2003). The metabolites, such as compound K, ginsenoside Rh2, and protopanaxatriol, have potent cytotoxicity against tumor cells, which may suggest the GM has a crucial role in exploiting the advantage of the bioactive compounds of ginsengs in full (Kim, 2018).

Importantly, the differences in GM composition among individuals is related to different metabolite outcomes. For instance, in a control setup, individuals who consume a fat- and protein-rich diet carry different microbes in the gut and, thus, higher concentrations of GF1 and GC-K metabolites were noticed after taking *Panax notoginseng* saponins. However, people who ate a fibrous diet had more GRh2, PPT, and PPD metabolites after taking *Panax notoginseng* saponins (Wang et al., 2021).

#### 2.2.2 GM can detoxify lower grade TCM

In “Sheng Nong’s herbal classic,” TCM is divided into three grades. Among them, the lower grade is more toxic, and it is often used after concocting to reduce its toxicity. It is recently reported that the GM can contribute to the detoxification of TCM. For instance, the GM lowers the toxicity of diester diterpene alkaloids by partly converting them to mono-ester aconitum alkaloids and lipo-alkaloids. Diester diterpene alkaloids are the main components of the radix aconiti, Kusnezoff monkshood, and Aconitum carmichali debx (Zhao et al., 2007; Wang et al., 2015; Yang et al., 2018). Another typical case is Baicalin, a glycoside present in *Scutellaria baicalensis Georgi*, which is converted to baicalein, a flavone with lower cytotoxic side effects (Khanal et al., 2012). However, the GM should be taken carefully in the context of reducing the toxicity of lower grade TCM. A host could inhabit certain bugs that could worsen the toxicity of a compound. The GM role has been suspected in elevating the toxicity of amygdalin that is extracted from *Armeniacae Amarae Semen* (Carter et al., 1980).

## 3 A complex interaction and collaboration between TCM–GM and Western medicine

In TCM classic theories, once TCM is combined, some will increase toxicity and some will reduce others’ efficacy (Zhou et al., 2017), which are the so-called 18 incompatible medicaments (*ShiBaFan*) and 19 medicaments of mutual restraint (*ShiJiuWei*) principles. To achieve a better curative effect and avoid side effects, combined TCM should be under the guidance of compatibility theories. Modern pharmacology reveals the rationality of the compatibility of TCM, besides this, from another angle, and GM modulation also explains the compatibility theory.

Even in the case of Western drugs, not all of them work for every patient, and a considerable number of patients are nonresponsive; this observation is known as nonresponse bias. And it is a growing consensus that the nonresponders are missing some important bacterial species in their gut. For instance, the efficacy of cyclophosphamide, an anticancer drug, is dependent on the presence of *Barnesiella intestinihominis* and *Enterococcus hirae* in the gut of a patient (Daillère et al., 2016). Not only GM but TCM has also been taken for consideration of enhancing the effectiveness of Western medicine. In one such case, a group of researchers found improved efficacy of the antiprogrammed cell death 1/programmed cell death ligand 1 (anti-PD-1/PD-L1) in the presence of ginseng polysaccharides. It was further confirmed that *Parabacteroides distasonis* and *Bacteroides vulgatus* were dominating the gut of the treated patient. Those patients who did not respond to the combinatorial treatment of the anti-PD-1/PD-L1 and ginseng polysaccharides had a depleted abundance of the *Parabacteroides distasonis* and *Bacteroides vulgatus* (Huang J. et al., 2021).

## 4 GM-based investigation of medicine’s efficacy – an exciting prospect for TCM–GM research

Nowadays, with the continuous development of culture, multi-omics combination and gene sequencing technologies, the exploration of TCM and microbiota is making exciting discoveries. The mechanism behind TCM theory about *Qi*, *Xue*, *Ying*, *Yang*, four properties and five flavors have received much attention. TCM regulates the composition and metabolites of intestinal flora, which can be regarded as one of the mechanisms for expounding the efficacy of TCM. We predict that one of the emerging research areas in TCM–GM research will be the investigation of the GM’s role in TCM’s efficacy. Particularly for those therapeutics that are taken orally. By taking the studies carried out on Western drugs as an example, here we try to explain how much the GM could contribute to the efficacy of the medicine. This could help TCM researchers to orient their research.

(1) As an example, we discuss a therapeutic approach that is based upon the development of antibodies to block CTLA-4, known as the cluster of differentiation 152 (a protein receptor working as an immune checkpoint), for the treatment of cancer. This technique is a current hot topic and has shown promising results in clinical trials. During this process, the body’s T cells are recruited against tumors that pose limited damage to the normal cells (Sivan et al., 2015). Preclinical studies have shown that the efficacy of anti-CTLA-4 therapy is dependent upon the composition of the GM. For instance, it has been discovered that anti-PD-L1 (an antibody that blocks CTLA-4) efficacy improves in the presence of *Bifidobacterium*. Oral administration of anti-PD-1 and *Bifidobacterium* has been observed with augmented dendritic cell function and improved CD8^+^ T cell priming in the tumor microenvironment (Sivan et al., 2015). Besides this, anti-CTLA-4 therapies have also shown dependence on the presence of *Bacteroides thetaiotaomicron* or *Bacteroides fragilis*. Nonetheless, the anti-CTLA-4 therapy has failed in germ-free mice and those treated with antibiotics (Vétizou et al., 2015). More recently, these preclinical experiments were reproduced in melanoma patients and it was found that patients who responded effectively to anti-CTLA-4 treatments were harboring enriched bacterial diversity belonging to the family *Ruminococcaceae* (Gopalakrishnan et al., 2018).

(2) As a second example, we discuss the efficacy of cyclophosphamide, an anticancer immune suppressant chemotherapeutic that also remodels the gut microbial composition. During cyclophosphamide treatment, the gram-positive bacteria translocate into the lymphoid organ and mimic the production of pathogenic T helper 17 cells and memory Th1 immune responses C. Especially, cyclophosphamide efficacy is dependent on two intestinal commensals known as *Barnesiella intestinihominis* and *Enterococcus hirae*. During cyclophosphamide therapy, *Barnesiella intestinihominis* accumulates in the colon and infiltrates γδT cells in the cancer lesion, whereas *Enterococcus hirae* translocates to secondary lymphoid organs and stimulates intratumoral CD8/Treg ratio (Daillère et al., 2016). Similarly, another study reports the interconversion of fluoropyrimidines (the first-line anticancer drug) by gut microbial vitamin B6, B9, and ribonucleotide metabolism (Scott et al., 2017). Microbial influences on the efficacy of chemotherapeutic drugs, 5-fluoro-2′-deoxyuridine, and 5-fluorouracil are also reported (García-González et al., 2017).

It is important to mention that microbiome-based therapies should be tailored to disease types and affected body sites. For example, men with metastatic prostate tumors who responded to checkpoint inhibition have been found to have lower levels of a microbe called *Akkermansia muciniphila* in their stool than men who did not respond. But the opposite is true of people with lung and kidney cancers: Those with more *A. muciniphila* in their guts tended to fare better on the therapy (Dolgin, 2020).

## 5 TCM–GM in mental disease

TCM has been used in treating neuropsychiatric diseases for thousands of years. Synopsis of Golden Chamber (a classic Chinese medicine book written by Zhang Zhongjing in the Han Dynasty) recorded a disease, Zang Zao, in which the main symptoms are depression of spirit, emotional disturbance, weeping and laughing hysterically. Although this kind of disease cannot be solved thoroughly, it can be alleviated effectively by the TCM formula Ganmai Dazao Decoction. Nowadays, lots of evidence uncovers TCM exerting alteration and restoration function of GM. In addition, the altered microbiome, through repairing the gut barrier, regulates the gut permeability, alleviating the inflammation and other potential mechanisms to cure or relieve the symptoms and disease. A gradual but strong link is establishing the connection of TCM, GM, and mental disease. For instance, it is demonstrated that TCM treatment of chronic unpredictable mild stress (CUMS) rats altered the abundance of *Ruminococcus* and *Roseburia* and potentially increased the expression of cysteine [83]. Meanwhile, N-acetylcysteine is considered beneficial for brain disorders [84].

In addition, it is reported that GM diversity has a strong association with insular resting state functional connectivity. A higher fecal bacterial microbiota diversity is linked to a higher resting state insular functional connectivity [78]. Interestingly, the fecal microbiota-derived indole metabolites are found to associate with functional and anatomical connectivity of the amygdala and anterior insular nucleus [79]. For example, *Bacteroides, Parabacteroides* and *Escherichia* species can promote production of the γ-aminobutyric acid (GABA). Particularly, a lower abundance of *Bacteroides* was found in depression patients [80]. A series of human mental diseases (such as posttraumatic stress disorder, bipolar disorder, anxiety, and stress, and so on) are associated with altered GM diversity [81].

Recent research shows that gut bacteria are directly sensed by hypothalamic neurons through bacteria muropeptides to regulate host feeding behavior. In this process, muropeptides are recognized by cytosolic Nod-like receptors, which are expressed by a subset of hypothalamic neurons. This subset also responds to muramyl dipeptide from the intestine, thus regulating food intake and associated behaviors [82]. It is revealed that Nod 2 mutation has a strong association with bipolar disorder, schizophrenia, and Parkinson’s disease [82].

## 6 Conclusion

TCM–GM interaction is a fertile research field for identifying the faulted signaling pathways during disease and finding a treatment for them by manipulating GM, and this could provide a modern framework for evaluation and validation of TCM. It is well-known that TCM exerts therapeutic abilities that are holistic in nature, which could be impractical to comprehend with conventional research tools. Therefore, we propose that the integration of TCM with GM research can target the wholeness of a biological system. In a few cases, the integration of GM with TCM and other natural products have already made landmark achievements in the field of diseases prognosis and treatment.

## Author contributions

WX and BL wrong a majjor part of the manuscript, ST collected the information, MY revised the manuscript, IK supervised the data and the manuscript. All authors contributed to the article and approved the submitted version.

## Funding

This project was funded by the Deanship of the Scientific Research (DSR), King Abdulaziz University, Jeddah, KSA, under the grant no. DF-244-141-1441.

## Conflict of interest

The authors declare that the research was conducted in the absence of any commercial or financial relationships that could be construed as a potential conflict of interest.

## Publisher’s note

All claims expressed in this article are solely those of the authors and do not necessarily represent those of their affiliated organizations, or those of the publisher, the editors and the reviewers. Any product that may be evaluated in this article, or claim that may be made by its manufacturer, is not guaranteed or endorsed by the publisher.

## Abbreviations

CRC, Colorectal Cancer; FXR, farnesoid X receptor; GABA, g-aminobutyric acid; GM, gut microbiome; PC, Pogostemon cablin; SCFAs, Short-chain fatty acids; TCM, Traditional Chinese Medicine; TJ, Tight Junction; TLR-4, M toll like receptor.
