# Corrigendum: The science behind TCM and Gut microbiota interaction-their combinatorial approach holds promising therapeutic applications

**DOI:** 10.3389/fcimb.2023.1302492

**Published:** 2023-10-11

**Authors:** Wenrui Xia, Bei Liu, Shiyun Tang, Muhammad Yasir, Imran Khan

**Affiliations:** ^1^ Hospital of Chengdu University of Traditional Chinese Medicine, Chengdu, China; ^2^ Chengdu University of Traditional Chinese Medicine, Chengdu, China; ^3^ National Drug Clinical Trial Agency, Teaching Hospital of Chengdu University of Traditional Chinese Medicine (TCM), Chengdu, China; ^4^ Special Infectious Agents Unit, King Fahd Medical Research Center, King Abdulaziz University, Jeddah, Saudi Arabia; ^5^ Department of Biotechnology, Abdul Wali Khan University Mardan, Khyber Pakhtunkhwa, Pakistan

**Keywords:** Chinese medicine, TCM, gut microbiota, prebiotics, TCM-microbiota interaction, TCM-bacteria interaction, medicine

## Incorrect affiliation

In the published article, there was an error in affiliation 1. Instead of “The First Affiliated Hospital of Chengdu University of Traditional Chinese Medicine, Chengdu, China”, it should be “Hospital of Chengdu University of Traditional Chinese Medicine, Chengdu, China”.

The authors apologize for this error and state that this does not change the scientific conclusions of the article in any way. The original article has been updated.

